# Evaluation of Physicochemical Properties, Antioxidant Activity, and Selenium Release Characteristics in Gastrointestinal Fluids of Microwave‐Assisted Selenized Chinese Angelica Polysaccharide

**DOI:** 10.1002/fsn3.71181

**Published:** 2025-11-10

**Authors:** Ruijuan Fan, Wanting Dai, Linqing Yue, Xiaoxiao Song, Yunpu Wang, Xian Cui

**Affiliations:** ^1^ State Key Laboratory of Food Science and Resources, China‐Canada Joint Lab of Food Science and Technology (Nanchang), Key Laboratory of Bioactive Polysaccharides of Jiangxi Province Nanchang University Nanchang China; ^2^ State Key Laboratory of Food Science and Resources, Engineering Research Center for Biomass Conversion, Ministry of Education Nanchang University Nanchang Jiangxi China

**Keywords:** antioxidant, Chinese angelica polysaccharide, microwave, Se release kinetics, selenium, selenylation polysaccharides

## Abstract

Selenium (Se) is an indispensable trace element for human health. Synthetic selenylation polysaccharides have emerged as promising sources of Se for dietary supplements. However, existing studies on the specific forms and valence states of Se in synthetic selenylation polysaccharides remain insufficient, which limits the study of their biological activities. Therefore, it is necessary to further investigate their biological activities by integrating these aspects. In this paper, selenylation Chinese angelica polysaccharide (Se‐CAP) was synthesized by microwave treatment. After Se modification, Chinese angelica polysaccharide (CAP) exhibited a diminished particle size, a reduced surface negative charge, and an increased molecular weight. Se incorporated into the CAP chain through O‐Se‐O and O‐H···Se, with a portion of Se^4+^ reduced to Se^0^. Concurrently, Se‐CAP synthesized with microwave power of 200 W at 70°C for 90 min, which was named Se‐CAP1, had the highest Se content of 13.22 mg/g, enhanced ABTS antioxidant activity of nearly 100% at 0.5 mg/mL, and HT‐29 cell viability of 158% at 0.1 mM. Furthermore, Se release kinetics revealed that Se‐CAP1 exhibited a Fickian release in the simulated gastric fluid and a non‐Fickian release in the simulated intestinal fluid. This study provides a reference for the optimization of the preparation of selenylation polysaccharides and a theoretical basis for the design and development of selenylation polysaccharides.

## Graphical abstract

Microwave‐assisted Se‐CAP was successfully achieved and reached an optimal selenium content of 13.22 ± 0.25 mg/g. Se‐CAP exhibited high antioxidant activity and low cytotoxicity. Se‐CAP exhibited a Fickian release in the simulated gastric fluid and a non‐Fickian release in the simulated intestinal fluid.

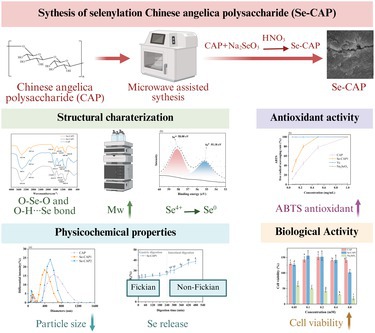

## 1 Introduction

Selenium (Se) is an indispensable trace element for human health and plays a pivotal role in disease prevention and treatment, including antiaging, antitumor, and antioxidative effects (Dai et al. 2025; Song, Chen, Sun, et al. 2020; Xiao et al. 2019). The multivalent nature of Se is the primary reason for its complex biological activity. Compared to organic Se and inorganic Se, zero‐valent Se possesses redox activity, superior free radical scavenging capacity, and lower toxicity (Moawad et al. 2024; Song et al. 2021; Song, Chen, Zhao, et al. 2020). However, its poor water solubility and low bioavailability make it unsuitable for direct use as a Se supplement (Cui et al. 2019).

Polysaccharides, which have high bioavailability and antioxidant activity (Aziz et al. 2024; Dai et al. 2023; Huang et al. 2023), tend to be selenized to synthesize selenylation polysaccharides, which are gaining increasing attention due to their low toxicity and high efficiency (Zhan et al. 2022). While some plants, animals, and microorganisms contain natural selenylation polysaccharides, their extremely low concentrations limit practical applications (Yue et al. 2022). In recent years, synthetic selenylation polysaccharides have emerged as promising Se supplements for their enhanced biological activities, including antioxidant, immune modulation, antitumor, iron‐chelating, and glucose‐lowering effects (Xiao et al. 2019). Although their biological functions are gradually being elucidated, existing studies on the specific forms and valence states of selenium within these compounds remain insufficient. It is necessary to further investigate their biological activities by integrating these aspects.

In previous studies, selenylation polysaccharides were predominantly prepared using hydrothermal methods. For instance, Guan and Zhao (2022) synthesized Se‐soluble yam polysaccharides using this approach, which resulted in enhanced immune activity. However, hydrothermal methods are limited by the prolonged reaction times (6–8 h), low Se content, and susceptibility of the polysaccharide structure to degradation (Wang et al. 2017). In recent years, the microwave‐assisted synthesis of selenylation polysaccharides has garnered attention for its high efficiency. The molecular vibrations under high‐frequency electric fields accelerate internal molecular movement, resulting in a significant reduction of synthesis time (Zeng et al. 2022). Moreover, selenylation polysaccharides prepared by microwave treatment exhibit an enhanced antioxidant activity, as exemplified by selenium polysaccharides from *Lonicera caerulea* L. fruits (Shao et al. 2023).

Chinese angelica (CA) is a renowned Chinese herbal medicine (Nai et al. 2021). Chinese angelica polysaccharide (CAP) is an important bioactive component from CA with immunomodulation, antiviral activities, antitumor effects, and antiphlogistic characteristics (Cheng et al. 2020). However, limited research has been conducted on the microwave‐assisted preparation of selenylation Chinese angelica polysaccharides (Se‐CAPs). Our previous research (Yue et al. 2022) revealed that the interaction between Se^4+^ and polysaccharides during microwave Se enrichment led to the conversion of Se^4+^ to Se^0^. Se^0^ is a vital valence of Se essential for selenylation polysaccharides to regulate immunity, maintain cardiovascular health, and prevent cancer. Thus, investigating the transition of the Se^4+^ oxidation state is important for enhancing the physiological activity of Se‐CAP, with potential implications for boosting its activity.

In this study, Se‐CAPs were prepared by the microwave‐assisted treatment via the HNO_3_‐Na_2_SeO_3_ reduction and characterized in structural properties, Se valence state changes, antioxidant activity, and biological activity. In addition, the Se release mechanism under simulated gastrointestinal conditions was investigated. This study offers an effective approach for selenylation polysaccharide preparation and provides a theoretical basis for the design and development of future products related to selenylation polysaccharides.

## 2 Materials and Methods

### 2.1 Reagents and Cell Culture

The CAP (98% purity) was sourced from Ci Yuan Biological (Shanxi, China), sodium selenite (Na_2_SeO_3_) from Xiya Chemical (Shandong, China), and acids from Xilong Science (Guangdong, China). 2,2‐Diphenyl‐1‐picrylhydrazyl (DPPH), 2,2‐Azinobis‐(3‐ethylbenzthiazoline‐6‐ sulphonate) (ABTS), phosphate buffered saline (PBS), ascorbic acid, pyrogallic acid, and the Cell Counting Kit‐8 (CCK‐8) were obtained from Macklin Chemical (Shanghai, China) and Dojindo (Tabaru, Japan). All other reagents were of analytical grade and obtained from Aladdin. HT‐29 cells were obtained from the national laboratory cell resource sharing platform and cultured in DMEM medium (Solarbio, China) supplemented with 20% fetal bovine serum (FBS; VivaCell, USA) at 37°C, 5% CO_2_.

### 2.2 Microwave‐Assisted Synthesis of Se‐CAP

Se‐CAPs were synthesized using a method previously reported (Yue et al. 2022). Briefly, 20 mL of 0.8% HNO_3_, 250 mg of CAP, and 200 mg of Na_2_SeO_3_ were mixed in a quartz tube. This tube was then placed into a microwave synthesizer (model DC8789, manufactured by CEM Co., City of Industry, CA, USA) and synthesized by adjusting the microwave parameters (temperature, time, and power). Se‐CAP1 and Se‐CAP2 were synthesized with microwave parameters of 70°C, 90 min, 200 W and 70°C, 60 min, 200 W, respectively. After microwave treatment, the solution was cooled to room temperature, and its pH was adjusted using sodium hydroxide. Next, excess Se nanoparticles were removed using a 3.0 kDa dialysis bag. Finally, the Se‐CAP product was obtained by freeze‐drying the dialysate.

### 2.3 Analysis of Physical and Chemical Properties of Se‐CAP

#### 2.3.1 Determination of Se and Polysaccharide Content

Se‐CAP was mixed with HNO_3_ and HCl, left to stand for 30 min, then microwaved in a microwave synthesizer (DC8789, CEM, USA) at 150°C for 10 min and subsequently at 180°C for 15 min. After cooling, the solution was heated to reduce volume, diluted to 100 mL with ultrapure water, filtered (mesh size of 0.22 μm), and analyzed for Se by inductively coupled plasma mass spectrometry (ICP‐MS; Agilent 7800, USA). The polysaccharide content was measured using the phenol‐sulfuric acid method.

#### 2.3.2 Fourier Transform Infrared Spectroscopy (FT‐IR)

5 mg CAP, Se‐CAP1, and Se‐CAP2 were mixed with 250 mg KBr, respectively. After grinding and pressing, their FT‐IR spectra (4000–400 cm^−1^) were measured using a Fourier Transform Infrared Spectrometer (FT‐IR; Nicolet iS50, Thermo, USA) with air as the reference (Zhang et al. 2025).

#### 2.3.3 Determination of Molecular Weight

The molecular weights of CAP, Se‐CAP1, and Se‐CAP2 were measured by gel permeation chromatography–high performance liquid chromatography (GPC‐HPLC; Waters, USA), employing ultrapure water containing 0.1 mol/L sodium nitrate as the flowing medium at a rate of 1 mL/min (Yuan et al. 2025).

#### 2.3.4 Measurement of Particle Size and Zeta Potential

Modified from Tian et al. (2023), a Zetasizer Nano ZS 90 (Malvern Instruments, UK) was used to measure particle size, polydispersity index, and zeta potential of CAP and Se‐CAP at 23°C ± 2°C. The solution was diluted to 0.625 mg/mL in ultrapure water with a refractive index of 1.333.

#### 2.3.5 X‐Ray Photoelectron Spectroscopy (XPS) and X‐Ray Diffraction (XRD) Analysis

The XPS spectra of Selenylation polysaccharides were acquired by X‐ray photoelectron spectrometer (Thermo Fisher Scientific, Waltham, MA, USA) to analyze the Se valency. Conditions: 1 × 10^−8^ Pa vacuum, Al Kα anode, 0–1000 eV range, 40 eV pass energy, 0.1 eV/step. Data fitting was performed using the XPSPEAK41 software. X‐ray diffractometer (D8 Advance, Bruker, Germany) was used to study crystal structure with a scanning range of 2θ = 5°–65°.

#### 2.3.6 Scanning Electron Microscopy (SEM) Analysis

The surface morphologies of the CAP, Se‐CAP1, and Se‐CAP2 were observed using a scanning electron microscope (JSM‐6701 F, Japan Electronics). The samples were attached to conductive glue, gold‐coated under vacuum, and imaged at 5.0 K and 20.0 K magnifications (Zhu et al. 2025).

### 2.4 Analysis of Se‐CAP Antioxidant Activity

The antioxidant activities of CAP and Se‐CAP1 against DPPH, ABTS, and superoxide anions were assessed, using vitamin C (Vc) as the positive control and Na_2_SeO_3_ as the negative control. Each experiment involved four sets of solutions: blank, positive control, sample, and sample control. Inhibition rate (%) was determined using the following Formula (1) (Yang et al. 2023):

(1) $$\text{Inhibition rate}\;\left(\%\right)=\left[1-\left(A_{1}\right)/A_{2}\right]\times 100\%$$

where A_0_ was the blank absorbance, A_1_ was the sample absorbance, and A_2_ was the sample reference absorbance.

For DPPH free radical determination, the sample solution was mixed with a 0.1 mmol/L DPPH ethanol solution at a 1:3 ratio, and a solution containing only ethanol served as the control. Following a 30‐min incubation period in the dark at room temperature, the absorbance was measured by a multifunctional microplate reader (Spectra Max 190, Molecular Devices, USA) at a wavelength of 517 nm.

For ABTS free radical determination, a solution of ABTS (7 mmol/L) was mixed with a solution of K_2_S_2_O_4_ (2.45 mmol/L), incubated in the dark for 16 h, and diluted using 5 mmol/L PBS (pH 7.4) until the absorbance reached approximately 0.70 at a wavelength of 734 nm. The sample, positive control, and blank groups were mixed with the ABTS working solution in a 1:4 ratio, while a sample solution containing only PBS served as the control. After 10 min of incubation at room temperature, absorbance was measured at 734 nm.

For superoxide anion radical determination, the sample solution was reacted with 4.5 mL Tris HCl (50 mmol/L, pH 8.2) for 20 min, then 0.3 mL pyrogallic acid was added. A control sample was prepared by replacing ultrapure water with pyrogallic acid. After incubation for 5 min at room temperature, the absorbance was measured at 320 nm.

### 2.5 In Vitro Simulated Gastrointestinal System Test

#### 2.5.1 Measurement of Se Release Characteristics

A 5 mg sample of Se‐CAP1 was combined with 10 mL of artificial gastric juice (pH 1.2) at 37°C and shaken at 200 r/min for a duration of 4 h. The pH was then adjusted to 7.4 using NaOH, followed by mixing with 10 mL of artificial intestinal fluid. The simulated samples underwent gastrointestinal digestion for durations ranging from 10 to 480 min. Subsequently, 0.4 mL of the digested sample was extracted and spun at 4°C and 10,000 rpm for 20 min (with a 3.0 kDa molecular weight cutoff) (Zhao et al. 2025). The ICP‐MS method was used to determine the Se content in the filtrate. The Se release rate (*R*_*t*_ %) at various time points (*t*) was calculated by the Formula (2), where *M*_*t*_ (mg) represents the amount of Se released at time *t*, and *M*_*0*_ represents the initial quantity of Se in the sample solution (Xiao et al. 2021).

(2) $$\;R_{t}\left(\%\right)=\frac{M_{t}}{M_{0}}\times 100$$

where *M*_*t*_ (mg) is defined as the quantity of Se released at a given time point (*t*), and *M*_*0*_ is the initial Se concentration present in the sample solution.

#### 2.5.2 Release Kinetics From Se‐CAP1

Se‐CAP1's Se release was analyzed with five kinetic models (Jana et al. 2013). The five equations are as follows:

1. Zero‐order equation, as shown in Formula (3)
(3) $$C=k_{0}t$$
2. First‐order equation, as shown in Formula (4)
(4) $$C=k_{1}\left[1-e^{\left(-\mathrm{kt}\right)}\right]$$

3 Higuchi equation, as shown in Formula (5)
(5) $$M=\mathrm{kt}0.5+k_{2}$$

4 Hixson‐Crowell equation, as shown in Formula (6)

(6) $$M={\left(k_{3}-\mathrm{kt}\right)}^{3}$$

where *M* represents the released active component at time *t*, *k* is the diffusion rate constant.

5 Korsmeyer‐Peppas equation, as shown in Formula (7)
(7) $$M_{t}/M_{\infty }=kt^{n}$$

where *k* and *t* represent the rate constant and time, respectively.

where *M* represents the released active component at time *t*, *k* is the diffusion rate constant.

where *M*_*t*_/*M*_*∞*_ represents sustained active component release, *t* is time, and *k* is the rate constant. The diffusion index *n* indicates the mechanism of Se release from Selenylation polysaccharides and the crosslinking between reactions. For spherical particles, the release mechanism is classified as follows: *n* ≤ 0.43 corresponds to Fickian release (case I transport); when 0.43 < *n* < 0.85, it is considered non‐Fickian release; *n* ≥ 0.85 indicates Fickian release (case II transport); and *n* > 1 represents super case II transport.

### 2.6 Effects of Selenylation Polysaccharides on the Activity of HT‐29 Cells (CCK‐8)

The CCK‐8 assay is based on the reduction of 2‐(2‐methoxy‐4‐nitrophenyl)‐3‐(4‐nitrophenyl)‐5‐(2,4‐disulfophenyl)‐2H‐tetrazolium sodium salt (WST‐8) to yellow methyl thiazolyl tetrazolium by mitochondrial dehydrogenase, with the reduction being proportional to the living cell count. A 96‐well plate was seeded with HT‐29 cells at a density of 1.2 × 10^5^ cells per well. A cell suspension of appropriate concentration was prepared and counted. Upon plate seeding, the cell suspension was mixed, and a blank group was established and incubated in a cell incubator for 12–24 h until cell attachment to the well surface was observed. Subsequently, the medium in each well was aspirated and washed 2–3 times with PBS. The experimental group received medium containing different sample concentrations, whereas the blank group received medium without samples. The 96‐well plate containing the HT‐29 cells was subsequently incubated within a cellular incubator under conditions at 37°C with a 5% CO_2_ atmosphere. Subsequently, 100 μL of 10% CCK‐8 solution was added to each well, and incubation continued until the optical density (OD) value reached approximately 1.0. The 96‐well plate was removed, and the final activity was determined using a multifunctional microplate reader (Spectra Max 190, Molecular Devices, USA) at 450 nm, with three repetitions of the measurements. The Formula (8) for the calculation is as follows:

(8) $$\text{Cell activity}\;\left(\%\right)=\left(A_{2}-A_{0}\right)/\left(A_{1}-A_{0}\right)\times 100$$

where *A*_*2*_ is the absorbance of cells, culture medium, CCK‐8 solution, and sample solution; *A*_*0*_ is the absorbance of (*A*_*2*_−*A*_cell_); *A*_*1*_ is the absorbance of (*A*_*2*_−*A*_sample solution_).

### 2.7 Statistical Data

The experimental data were measured three times and expressed as mean ± standard deviation. Statistical analysis was performed using one‐way ANOVA followed by Tukey's post‐test via SPSS 22.0. Significance was determined at *p* < 0.05.

## 3 Results and Discussion

### 3.1 Single‐Factor Experiment of Selenylation Modification

**FIGURE 1:**
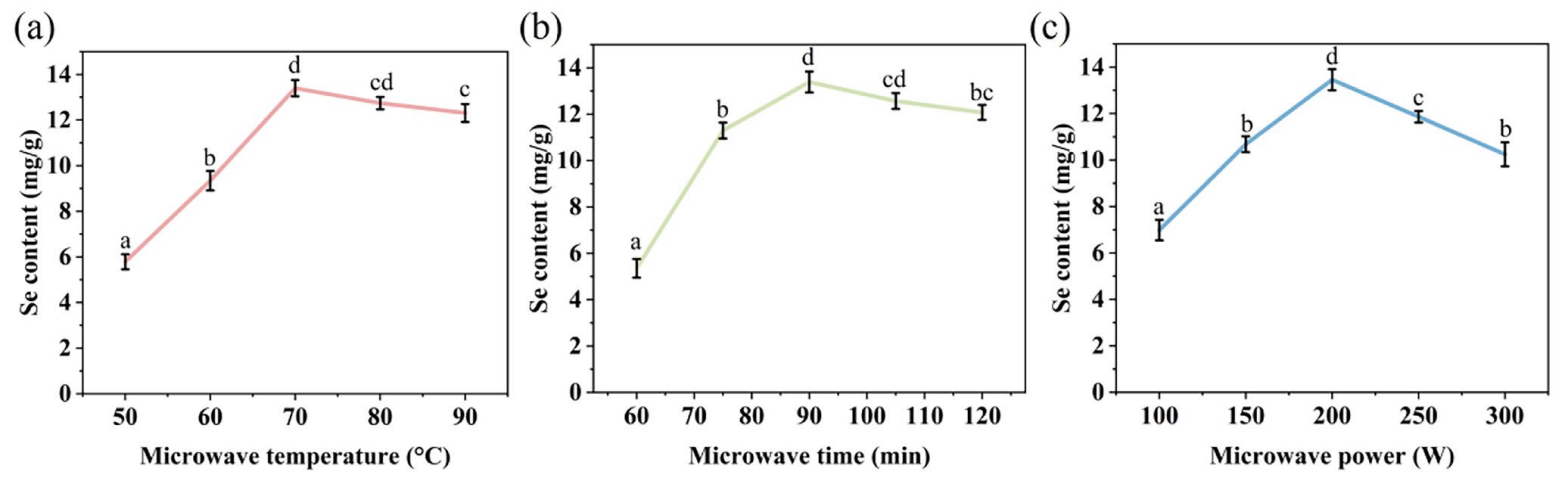
Se content in Se‐CAP prepared by microwave treatment with different temperatures, times, and power. (a) Se content in Se‐CAP with different microwave temperatures at 90 min and 200 W; (b) Se content in Se‐CAP with different microwave times at 70°C and 200 W; (c) Se content in Se‐CAP with different microwave power at 70°C and 90 min. Different letters indicate significant differences at *p* < 0.05 level. *n* = 3.

The effects of different microwave temperatures, reaction times, and power levels on the Se content in Se‐CAP were investigated using the single‐factor experiment to optimize the Se‐CAP preparation. The results in Figure 1 indicated that the Se content in Se‐CAP initially increased and then decreased as temperature, time, and power increased, which was similar to the findings in the microwave‐assisted synthesis of selenylation *Potentilla anserina L*. polysaccharides (Zhao et al. 2013). Figure 1a showed that, at a microwave power of 200 W and a reaction time of 90 min, the Se content in Se‐CAP increased as the temperature rose and reached a maximum of 13.22 mg/g at 70°C. Thus, 70°C is determined to be the optimal reaction temperature. Figure 1b demonstrated the effect of reaction times on Se content under the condition of 200 W and 70°C. When the reaction time was between 60 and 90 min, the Se content increased significantly with time, and reached a maximum at 90 min. Figure 1c indicated that, under the conditions of 90 min, 70°C, and 200 W, the Se content peaked at 13.22 mg/g. However, when the temperature, time, and power exceeded the optimal conditions, the Se content began to decrease. This decline may be due to the intensified polysaccharide degradation caused by higher temperatures and power, as well as longer reaction times, which inhibited the incorporation of Se into the polysaccharide (Wei et al. 2015).

The optimal reaction conditions were determined to be a temperature of 70°C, a reaction time of 90 min, and a microwave power of 200 W, under which Se‐CAP with the highest Se content (13.22 mg/g) was obtained and named Se‐CAP1. Compared to the Se‐CAP synthesized by Qin et al. (2013) using a hydrothermal method at 70°C for 480 min, the microwave method significantly reduced the reaction time (90 min), demonstrating its advantages in the synthesis of selenylation polysaccharides. Meanwhile, the polysaccharide with the lowest Se content (5.56 mg/g, 200 W, 70°C, 60 min) was named Se‐CAP2. To improve both economic viability and efficiency, Se‐CAP1 and Se‐CAP2 will be used for subsequent physicochemical characterization and activity analysis.

### 3.2 Structural Analysis of Se‐CAP

#### 3.2.1 SEM Analysis

**FIGURE 2:**
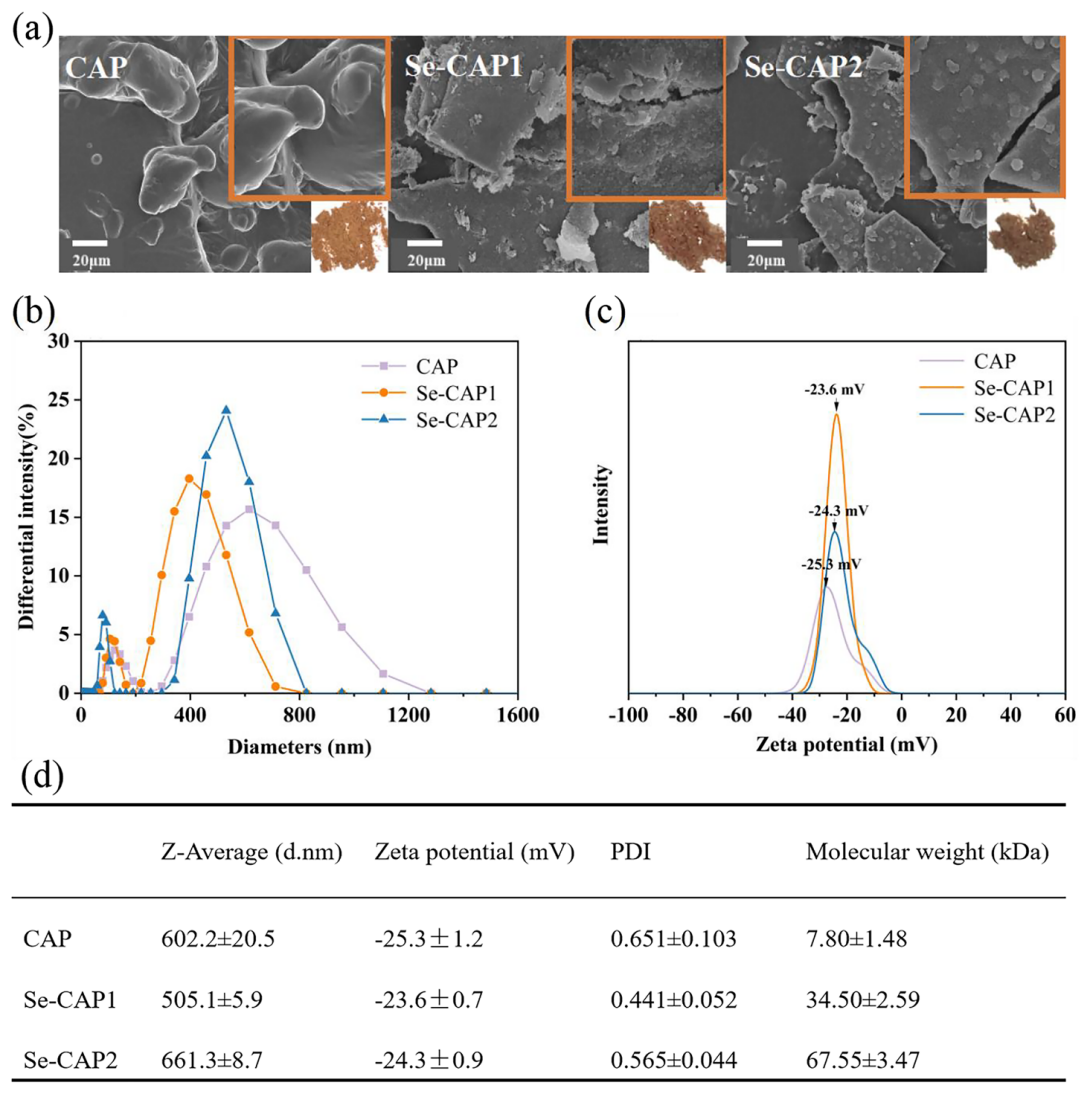
Characterization of CAP, Se‐CAP1, and Se‐CAP2. (a) SEM images, physical images, (b) particle size, and (c) zeta potential distribution of CAP, Se‐CAP1, and Se‐CAP2. (d) Results of particle size, potential, PDI and molecular weight of CAP, Se‐CAP1 and Se‐CAP2. Inserted figures in the bottom right corner of SEM images were the physical images. Scale bar: 20 μm. Se‐CAP1 and Se‐CAP2 were synthesized with microwave parameters of 70°C, 90 min, 200 W and 70°C, 60 min, 200 W, respectively. *n* = 3.

The surface microstructure and physical images of CAP, Se‐CAP1, and Se‐CAP2 were illustrated in Figure 2a. It was observed that the microstructure of CAP underwent significant changes after Se modification of CAP by microwave. The CAP exhibited a smooth surface and an irregular spherical and cross‐linked framework. However, the spherical structure of CAP after Se modification was disrupted, resulting in a sheet‐like form with an increased specific surface area, darker color, and smaller particle size. This may be due to changes in the interaction forces between polysaccharide molecules during Se modification, which is consistent with previous reports by Shao et al. (2023) and Lee et al. (2018). The alterations in the molecular structure of polysaccharides induced by selenylation were likely due to modifications in intramolecular and intermolecular hydrogen bonds, as well as van der Waals forces (Zhan et al. 2022). Furthermore, upon the comparison between Se‐CAP1 and Se‐CAP2, it was noted that the former, with a longer microwave time, exhibited a rougher surface. In contrast, the latter displayed a smoother surface, albeit with attached particles. Furthermore, the particles attached to the surface of Se‐CAP1 were clear and low in density, whereas the particles on the surface of Se‐CAP1 were densely packed together, making them more prominent. This phenomenon may be attributed to the binding and aggregation of Se atoms on the CAP surface, altering its surface morphology (Yang et al. 2023). Moreover, as the amount of bound Se increased, the surface modification of Se‐CAP became more significant. Additionally, in the bottom right corner of each image, there was a corresponding physical representation of CAP. Notably, the second physical representation appeared reddish, suggesting the possible formation of red amorphous Se.

#### 3.2.2 DLS Analysis

The particle size distribution, zeta potential, and polydispersity index (PDI) of CAP, Se‐CAP1, and Se‐CAP2 were analyzed using the DLS method, and the obtained results are presented in Figure 2b–d. With the increase of Se‐rich content in CAP, the PDI of CAP gradually decreased and the minimum PDI of Se‐CAP1 was 0.441. Due to smaller PDI, the polysaccharide particles distributed more uniformly in the hydrated solution. Therefore, Se‐CAP1 exhibited a more uniform distribution in solution (Sun et al. 2023). Additionally, Se‐CAP1 exhibited a decreased particle size compared to CAP, measuring 505.1 ± 5.9 nm. The findings indicated that microwave conditions facilitated the Se modification of CAP, resulting in the binding and coiling of CAP chains within Se‐CAP, and ultimately leading to more evenly dispersed polysaccharide particles in the hydrated solution. As shown in Figure 2c, the absolute value of the zeta potential of CAP diminished as the Se content increased, suggesting that the degree of hydration of the negative surface charges on Se‐modified CAP was reduced under microwave action, leading to a decreased dispersion of Se‐CAP in the solution. This finding was aligned with the results obtained by Yue et al. (2022), indicating that microwave non‐thermal effects influenced the deprotonation process of ionizable groups present on the surface of CAP during selenization modification.

#### 3.2.3 Molecular Weight Analysis

The molecular weights of CAP, Se‐CAP1, and Se‐CAP2 were determined by GPC. The results in Figure 2d showed that the molecular weights of Se‐CAP1 (34.50 kDa) and Se‐CAP2 (67.55 kDa) were significantly higher than that of CAP (7.80 kDa). Furthermore, although the Se content of Se‐CAP2 was lower than that of Se‐CAP1, its molecular weight was higher. This phenomenon resembled the findings of Zhao et al. (2013), which suggested that structural changes in CAP molecules during microwave‐assisted selenylation might reduce the relative density of CAP with increasing Se content, thereby affecting its molecular weight. In addition, microwave irradiation played a crucial role in selenylation polysaccharides synthesis. Microwave energy interacted with molecules at an extremely rapid rate, facilitating molecular coupling through selective absorption. This mechanism not only enhanced the efficiency of Se coupling with CAP molecules but also significantly increased the molecular weight of selenylation polysaccharides (Wang et al. 2017). In contrast, selenylation polysaccharides prepared using hydrothermal methods tended to have lower molecular weights (Wei et al. 2015). Zhang et al. (2011) further demonstrated that microwave effects polarized polar bonds, including C‐O‐C glycosidic bonds, thereby enhancing molecular reactivity. During this process, the generated Se^0^ might induce aggregation in the polysaccharide chain structure through a mediating effect and ultimately increase its relative molecular weight. This discovery was not only aligned with our molecular weight measurements but also validated the significant role of microwave technology in boosting efficiency in polysaccharide derivatives and copolymer synthesis (Mirzadeh et al. 2020).

#### 3.2.4 FT‐IR Analysis

**FIGURE 3:**
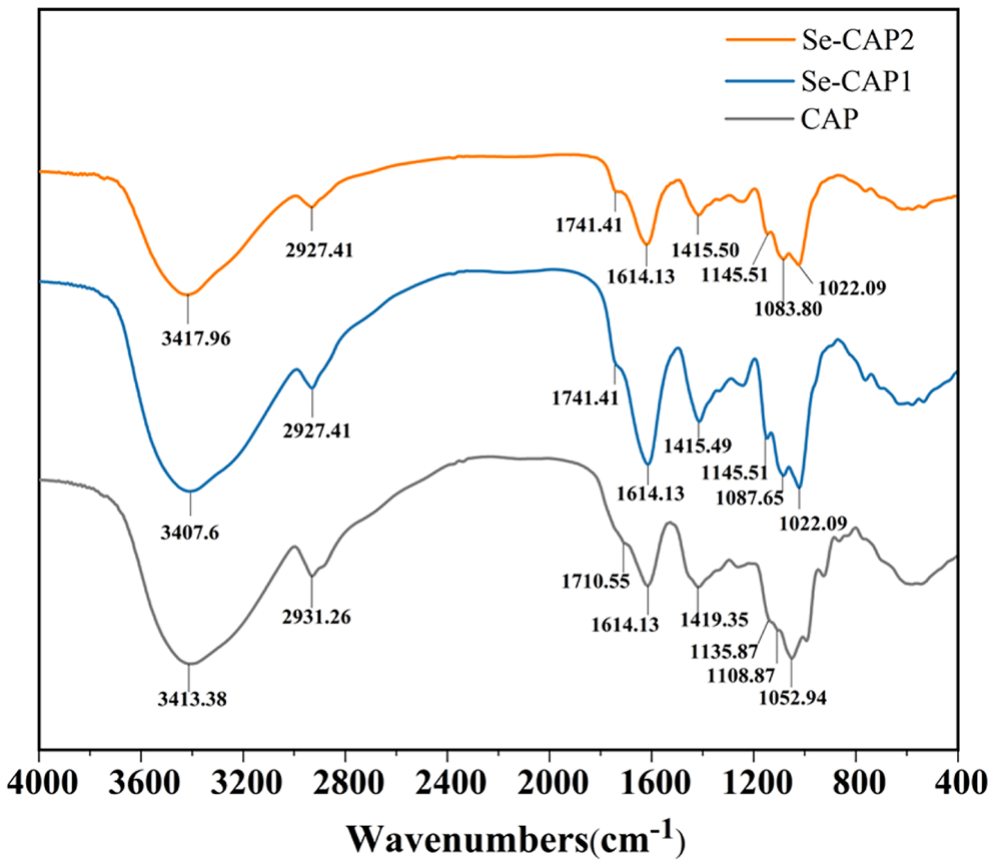
FT‐IR spectra of CAP, Se‐CAP1, and Se‐CAP2. Se‐CAP1 and Se‐CAP2 were synthesized with microwave parameters of 70°C, 90 min, 200 W and 70°C, 60 min, 200 W, respectively. *n* = 3.

The infrared spectra of CAP, Se‐CAP1, and Se‐CAP2 are depicted in Figure 3. All three polysaccharides exhibit a broad and strong absorption peak at 3200–3600 cm^−1^, indicating the stretching vibration of the ‐OH group. The weak absorption peak observed at 2927 cm^−1^ in all three spectra corresponds to the C‐H vibration of methyl and methylene groups. The vibrational stretching of the Se‐CAP carboxyl C=O at 1741.41 cm^−1^ exhibits a blueshift compared to CAP (1710.55 cm^−1^), indicating the possibility of the carbonyl undergoing a selenylation reaction. The FT‐IR spectra of Se‐CAP closely resembled that of CAP, demonstrating that the fundamental structure of CAP was unaltered after Se modification.

The ‐OH stretching vibration peak of Se‐CAP1 exhibited a shift compared to that of CAP (from 3413.38 cm^−1^ to 3407.60 cm^−1^), which was possibly due to changes in the spatial position of the Se, altering the hydroxyl state involved in molecular hydrogen bonding, with infrared evidence of the O‐H···Se bond (Surhio et al. 2017). In contrast, the shift on the ‐OH stretching vibration peak of Se‐CAP2 was less significant (from 3413.38 cm^−1^ to 3417.96 cm^−1^), demonstrating a smaller amount of O‐H···Se bond in Se‐CAP2. The higher content of O‐H···Se bond in Se‐CAP1 indicated the increasing Se enrichment during the microwave time from 60 min to 90 min. Additionally, the new absorption peak at 1022.09 cm^−1^ in Se‐CAP1 and Se‐CAP2 spectra indicated the presence of O‐Se‐O linkage (Li et al. 2017). These results indicated that Se modification of CAP was achieved through O‐Se‐O and O‐H···Se bonds. Furthermore, Se‐CAP1 and Se‐CAP2 both displayed two characteristic absorption peaks of the pyran ring within the range of 1155–1029 cm^−1^, which were credited to stretching vibrations of O‐C‐O and C‐O‐H. Compared to CAP (1135.87 cm^−1^ and 1108.87 cm^−1^), the peaks of Se‐CAP1 (1145.51 cm^−1^ and 1087.65 cm^−1^), and Se‐CAP2 (1145.51 cm^−1^ and 1083.80 cm^−1^) showed significant shifts, which may be due to the enrichment of Se causing changes in the absorption spectrum peaks of glycosidic bonds within the pyranosyl ring. Therefore, the molecular structures of both Se and CAP were affected by their combination (Zhai et al. 2024).

#### 3.2.5 XPS and XRD Analysis

**FIGURE 4:**
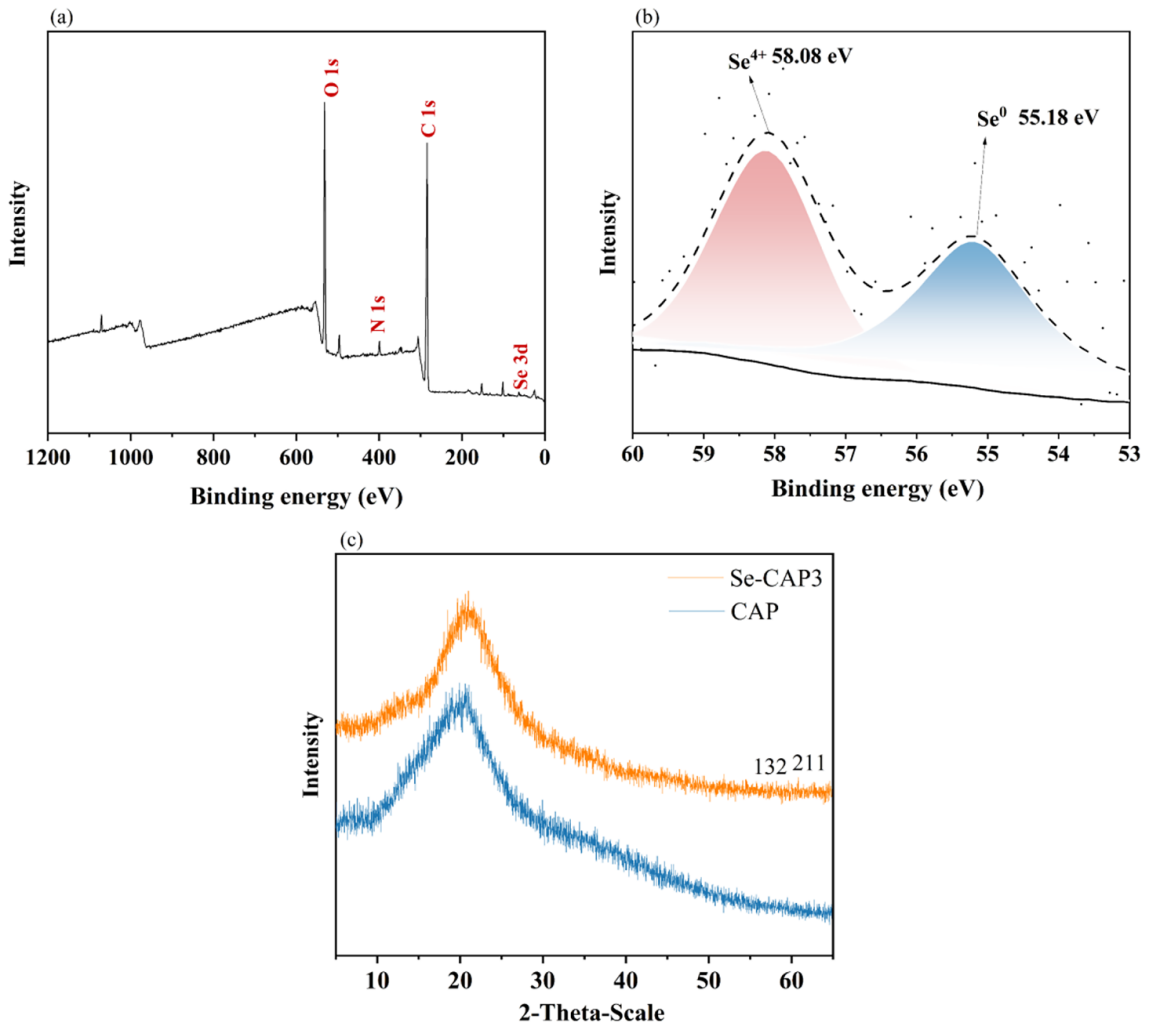
(a) Wide‐range XPS patterns and (b) Se 3d spectra of Se‐CAP1; (c) XRD pattern of CAP and Se‐CAP1. *n* = 3.

The XPS method was used to investigate the valence state composition of Se in Se‐CAP1, and the results were shown in Figure 4a,b. It was observed that Se‐CAP was primarily composed of Se, N, C, and O elements, with a C/O ratio of 2.5. The spectral fitting outcomes in the 3d region of Se revealed the observation of a distinct peak for Se^0^ at 55.18 eV, while a signature peak for Se^4+^ was detected at 58.08 eV with a peak area ratio of 1.1. This indicated that after microwave Se modification, Se‐CAP exhibited the coexistence of 0‐valent Se and +4‐valent Se. This phenomenon arose because the microwave radiation enhanced the conductive mobility of dissolved ions in the reaction mixture, which accelerated the transformation of SeO_3_^2−^ (selenite salt) to Se^0^ (Se) in Se‐CAP, thereby promoting the generation of Se^0^ (Shen et al. 2022). Furthermore, the crystal structure of Se in CAP and Se‐CAP1 was examined using XRD, and the outcomes were depicted in Figure 4c. According to the JCPDS (41‐0259) database, Se‐CAP1 exhibited an absorption peak at 59.682° (132), representing the diffraction peak of H_2_SeO_3_ (Se^4+^). Additionally, in the JCPDS (47‐1515) database, an amorphous diffraction peak of Se^0^ was identified at 60.831° (211), confirming the presence of Se^4+^ and Se^0^ in Se‐CAP1. In summary, the results from XRD and XPS analysis revealed that the primary forms of Se in Se‐CAP1 were Se^4+^ and Se^0^.

### 3.3 Antioxidant Capacity

**FIGURE 5:**
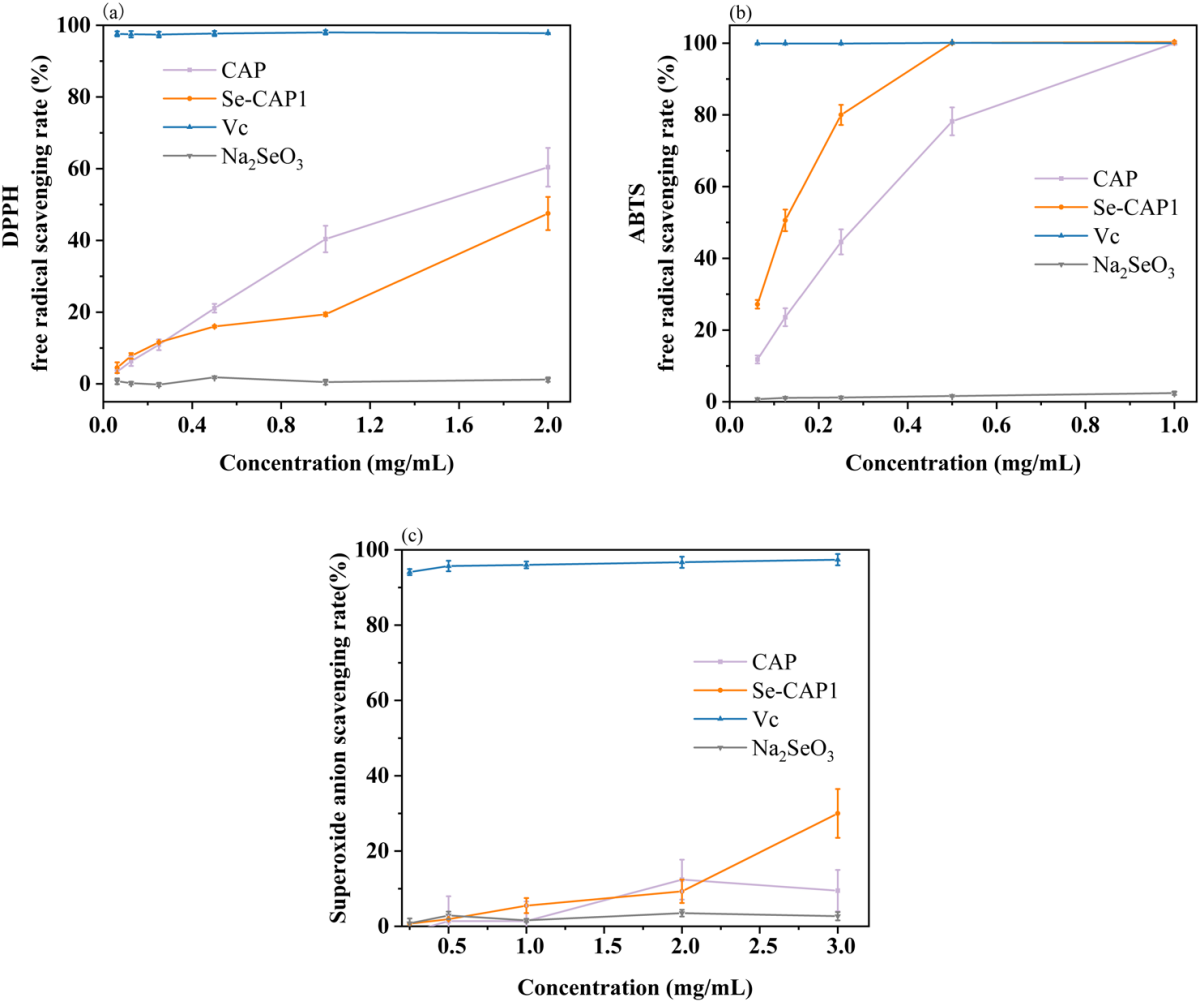
Antioxidant efficacy of CAP and Se‐CAP1. (a) DPPH radical scavenging activity. (b) ABTS radical scavenging activity. (c) Superoxide radical scavenging activity. *n* = 3.

Figure 5 depicts the variations in the scavenging capabilities exhibited by DPPH, ABTS, and superoxide anion radicals among CAP, Se‐CAP1, Na_2_SeO_3_, and Vc. In Figure 5a, within the concentration interval ranging from 0.25 to 2.0 mg/mL, the scavenging potency of CAP for free radicals outstripped that of Se‐CAP1, with respective IC50 values of 1.359 mg/mL and 3.264 mg/mL (*p* < 0.05). It was consistent with the findings of Wang et al. (2012a, 2012b), which revealed a lower DPPH scavenging activity after selenized modification of *A. sphaerocephala* polysaccharide. However, this observation contrasts sharply with prior conclusions positing a superior DPPH scavenging activity for Se‐modified rice embryo polysaccharides compared to their original counterparts (Chen et al. 2019). This phenomenon might be due to the strong ability of CAP to scavenge DPPH free radicals. The particle size of CAP decreased, and its molecular weight increased after microwave Se modification, resulting in a denser polysaccharide structure, hindering the ability of DPPH free groups in it and in the solution. However, Se‐CAP1 retained a higher scavenging activity compared to +4 valent Se (Na_2_SeO_3_). At a concentration of 2.0 mg/mL, the clearance rate of Se‐CAP1 exceeded that of Na_2_SeO_3_ by approximately 46.9%.

As shown in Figure 5b, the scavenging capabilities of Se‐CAP1 and CAP towards ABTS radicals were concentration‐dependent. Specifically, at a concentration of 0.500 mg/mL, the scavenging rate of Se‐CAP1 reached nearly 100%, with a calculated IC50 value of 0.184 mg/mL. In contrast, CAP achieved a scavenging rate of approximately 78.2% at the same concentration, with an IC50 value of roughly 0.451 mg/mL. This disparity could be attributed to the Se groups in the selenylation polysaccharides catalyzing the hydrogen atoms of isomerized carbons, thereby intensifying their ability to combat ABTS radicals (Gao et al. 2020). It was consistent with the findings of Shao et al. (2023), which revealed a higher ABTS scavenging activity after selenized modification of polysaccharides from *Lonicera caerulea L. fruits*. Furthermore, at a concentration of 0.5 mg/mL, the ABTS antioxidant activity of Na_2_SeO_3_ was merely 1.6%, significantly lower than the near‐perfect scavenging efficiency demonstrated by Se‐CAP1. This may stem from the substantial enhancement in the antioxidant activity conferred by the transformation of the oxidation state of Se from Se^4+^ to Se^0^ in Se‐CAP1.

In Figure 5c, within the concentration range of 0–2.000 mg/mL, the superoxide anion scavenging rates of CAP and Se‐CAP1 increased gradually with increasing concentrations, mirroring a similar trend exhibited by Se‐rich polysaccharides in *Brassica juncea* (Xiang et al. 2022). However, as the concentration reached 3.000 mg/mL, the superiority of Se‐CAP1 in scavenging superoxide anions became more evident, achieving a scavenging rate of 30%, whereas CAP lagged at 9.5%. The 1C50 values were 5.124 and 10.280 mg/mL, respectively. This divergence might be attributed to structural alterations in selenylation polysaccharides within Se‐CAP1 owing to the formation of linkages between Se groups (Se‐H) and selenate (O‐Se‐O), resulting in an increased abundance of hydroxyl groups and subsequently bolstering the antioxidant prowess of polysaccharides (Wei et al. 2015). Moreover, at an identically high concentration of 3.000 mg/mL, the scavenging rate of Na_2_SeO_3_ was merely 2.7%, paling in comparison to the formidable scavenging ability of Se‐CAP1. This observation suggested that the amount of Se in the elemental state (Se^0^) of Se‐CAP1 was positively related to its resistance against oxidation, as demonstrated by *Astragalus* selenylation polysaccharides containing Se^0^, which exhibited an enhanced antioxidant activity (Yue et al. 2022). Additionally, Se might act as a catalyst to activate the active centers of Se‐dependent antioxidant enzymes in biological systems, thereby enhancing their antioxidative activities (Sun et al. 2023).

### 3.4 Simulated Gastrointestinal In Vitro Release Profiles

#### 3.4.1 In Vitro Release Behavior of Se‐CAP1 in Digestive Conditions

**FIGURE 6:**
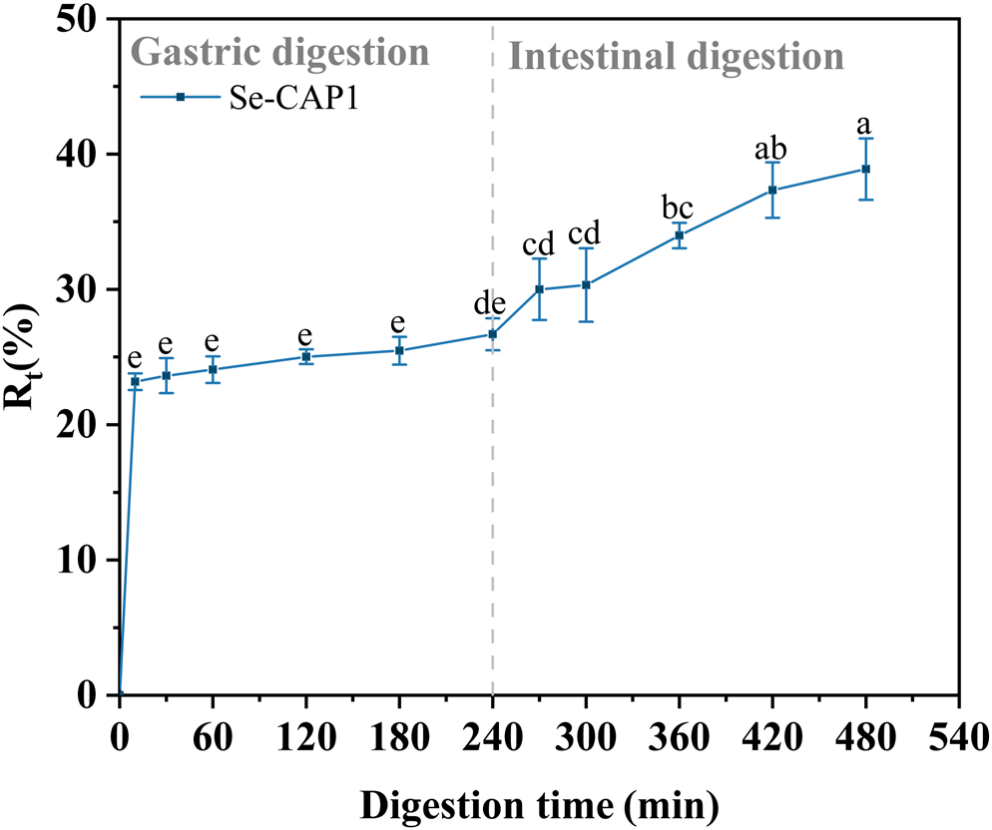
Se release rates of Se‐CAP1 in simulated gastrointestinal digestion. Different letters indicate significant differences at *p* < 0.05 level. *n = 3*.

The National Institutes of Health (NIH) recommended that the allowable daily intake of Se for adults should be within the range of 55–400 μg, indicating the narrow marginal dose of nutritional toxicity (Gu and Gao 2022). Therefore, it is essential to study the release characteristics and mechanisms of Se in the gastrointestinal environment for the application of Se supplements. In Figure 6, the release curve of Se‐CAP1 during the digestion of the stimulated gastric fluid stage (SGF, pH 1.2) and stimulated intestinal fluid stage (SIF, pH 7.4) is displayed, showing a time‐dependent release pattern and an increasing trend in the total release of Se. During the SIF stage, the release rate of Se‐CAP1 increased by 12.21%, reaching 38.89% ± 2.27% at 8 h. This indicated that Se‐CAP1 exhibited enhanced bioavailability in the SGF stage. Additional investigations revealed a swift acceleration in the release rate of Se during the initial 10 min, reaching a relatively high level at 10 min. The “burst effect” may have occurred in the first 10 min, considering the initial Se concentration of 0 in the environment. The “initial burst” stage in Se‐CAP1 accounted for 13.1% of the total Se release rate during the SGF stage. At this stage, Se‐CAP1 was initially destroyed by pepsin and strong acid in the SGF, resulting in the rapid release of Se (Yang et al. 2023). Then, CAP was degraded by trypsin in the SIF, resulting in an increased release of Se (Zhang et al. 2022).

#### 3.4.2 Controlled‐Release Kinetics of Se‐CAP1

**FIGURE 7:**
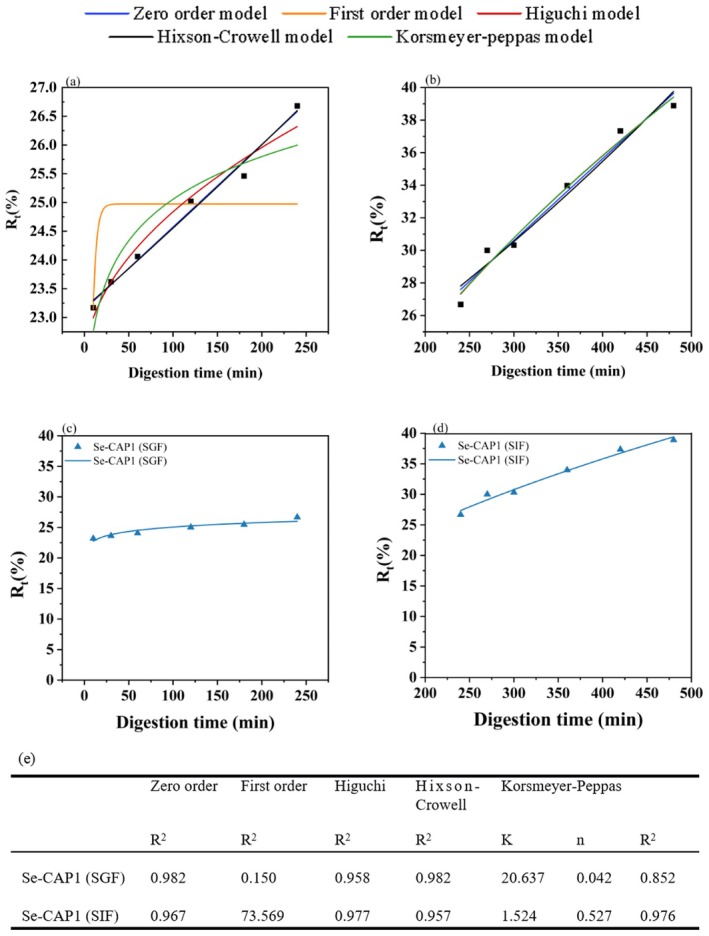
Se release kinetics analysis. (a, b) Se release kinetics of Se‐CAP1 under simulated digestive conditions. (c, d) The fitting data of Se release rates of Se‐CAP1 in (c) SGF and (d) SIF using the Korsmeyer‐Peppas model. (e) Comparison of Se release kinetic parameters of under different mathematical models. *k*, rate coefficient in the Korsmeyer‐Peppas model; *n*, kinetic release exponent in the Korsmeyer‐Peppas model; *R* ^2^, coefficients of correlation specific to individual kinetic models.

In order to further determine the Se release mechanism, five classical release models (Section 2.5.2) were utilized to evaluate the Se release mechanism of Se‐CAP1 under SGF and SIF conditions, as shown in Figure 7a,b. The parameters for the five models are presented in Figure 7e. The best‐fitted model was selected based on the correlation coefficient (*R* ^2^). The Zero‐order model emerged as the optimally fitted model for the Se‐CAP1 release pattern during the SGF phase. However, its adjusted *R* ^2^ was only 0.0003 higher than that of the Hixon‐Crowell model. Therefore, the Hixon‐Crowell model was also considered the optimal matching. In the SIF phase, the best‐performing model was the Higuchi model (*R* ^2^ = 0.977), followed by the Korsmeyer‐Peppas model, with an *R* ^2^ value of 0.976, which was only 0.001 lower than the Higuchi model. The Zero‐order model was the best‐fitted model for the Se release behavior of Se‐CAP1 in the SGF phase, indicating a controllable release of Se from the Se‐CAP1 (Nayak and Pal 2011).

Further, the Korsmeyer‐Peppas model was utilized to reveal the Se release mechanisms: Fickian diffusion release (*n* ≤ 0.43), case II transport (*n* ≥ 0.85), and the intermediary non‐Fickian release mechanism (0.43 < *n* < 0.85). The Se release rate in the SGF and SIF was fitted well by the Korsmeyer‐Peppas model with an *R* ^2^ value of 0.852 and 0.976, respectively, as shown in Figure 7c–e. The *n* value for Se release during the SGF stage was 0.042, which is below 0.43. Therefore, the release of Se in the acidic fluid was predominantly governed by both diffusion and expansion, which was assigned to the category of Fickian diffusion behavior, also known as case I transport (Nayak and Pal 2011). As shown in Figure 7e, the k value of Se‐CAP1 during the SGF (pH = 1.2) stage significantly exceeded that observed during the SIF (pH = 7.4) stage, with a difference of 19.113. This suggests that the rate of Se‐CAP1 release is higher under acidic conditions compared to neutral conditions, which was potentially due to the swelling and partial breakdown of CAP in the solution. With the extension of the dissolution duration, the disintegration of the polysaccharide polymer led to the continuous penetration of the acidic solvent, resulting in an erosion/degradation phenomenon and further destruction of the selenylation polysaccharide particles (Xiao et al. 2021). In summary, the swelling rate of Se‐CAP1 was lower than the erosion rate in the SGF stage, indicating a diffusion mechanism. The n value of Se release in the SIF stage was 0.527 (0.43 < 0.527 < 0.85) (Figure 7e). The swelling rate of Se was equal to the erosion rate, which followed a non‐Fickian release mechanism, indicating the simultaneous existence of diffusion and erosion (Jana et al. 2013). In addition, microwave irradiation had some effects on the Se in Se‐CAP1. In the gastrointestinal environment, Se^0^ and Se^4+^ present in Se‐CAP1 prepared via microwave can diffuse as soluble active components. Meanwhile, CAP swells and erodes in the simulated digestive system (Maderuelo et al. 2011). In addition, external aqueous solvents can infiltrate the polysaccharide structure and cause swelling. Meanwhile, the association bonds between Se and polysaccharides in Se‐CAP1 are broken. Therefore, Se easily permeates the macromolecular chains and escapes from the polysaccharide structure.

Figure 7c,d illustrated the release effect of Se‐CAP1 in SGF and SIF fitted by the Korsmeyer–Peppas model. Initially, the release rate of Se from Se‐CAP1 rapidly climbed to 22.5% during the early stages of SGF exposure. Subsequently, the release rate of Se changed minimally. In the SIF stage, the release rate increased, which was aligned with the actual in vitro release characteristics of Se‐CAP1 as previously concluded. Based on the n‐value analysis of Korsmeyer–Peppas's model discussed earlier, the low initial Se concentration in the simulated environment of digestive secretions contributed to the observed “initial burst”. However, as the internal Se diffused outwards over time with a gradual increase in Se concentration, the release rate of Se was suppressed. Consequently, a gradual release of Se was detected in the SGF stage, which was characterized by Fickian diffusion. As the selenylation polysaccharides expanded, the closely intertwined polysaccharide chains shifted to a more unconstrained configuration with increased fluidity. This allowed Se to diffuse through the swollen layer. Ultimately, the degradation within the swollen polysaccharide structure led to the release of residual Se, enhancing the release velocity during the SIF period (Xiao et al. 2021).

### 3.5 Examination of the Impact of Selenylation Polysaccharides on the Activity of HT‐29 Cells

**FIGURE 8:**
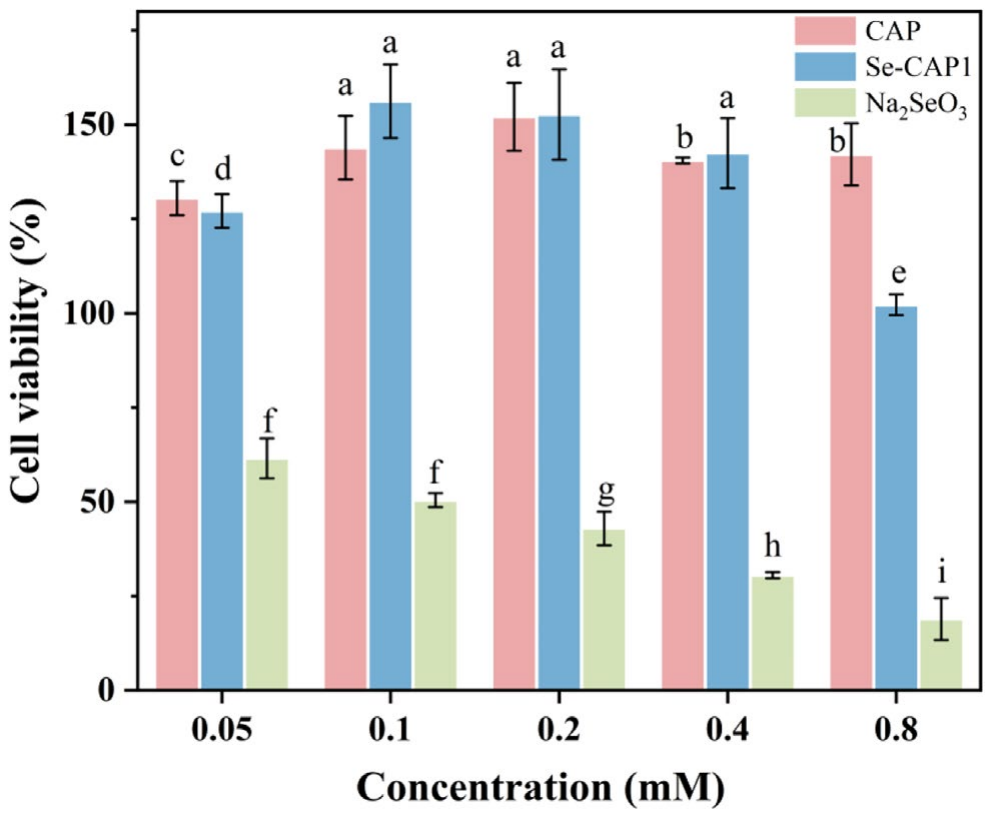
Impact of CAP and Se‐CAP1 on the viability of HT‐29 cells. Different letters indicate significant differences at *p* < 0.05 level. *n* = 3.

The impact of varying doses of CAP, Se‐CAP1, and Na_2_SeO_3_ on the activity of HT‐29 cells was assessed using the CCK‐8 method, and the results were shown in Figure 8. The different HT‐29 cell activity when applying various doses of CAP, Se‐CAP1, and Na_2_SeO_3_ indicated that their effects on the cell survival rate were dose‐dependent. In the concentration range of 0.05–0.8 mM, CAP and Se‐CAP1 exhibited similar trends in cell activity. With an increased concentration, the activity of HT‐29 cells exhibited an initial increase followed by a decrease. When 0.1 mM Se‐CAP1 was applied to HT‐29 cells, the survival rate reached 156.21%, which was 12.4% higher than CAP (143.8%). However, as the concentration gradually increased, its activity decreased in a dose‐dependent manner. At a concentration of 0.8 mM, the activity of HT‐29 cells decreased, and the survival rate of cells treated with Se‐CAP1 was 22.29% lower than that of CAP. In the corresponding concentration range, CAP and Se‐CAP1 exhibited higher effects on promoting cell activity, which were substantially greater than the effects of Na_2_SeO_3_ in the control group, achieving statistical significance at *p* < 0.05. This indicated that CAP itself had high nutritional characteristics and that Se‐modified CAP showed higher nutritional activity, promoting cell growth, and enhancing cell activity. Upon exceeding a dose of 0.1 mM, the toxicity of Se^4+^ accumulated slowly, and the suppressive action of Se‐CAP1 on cell activity gradually became apparent, further confirming the narrow nutritional‐toxicity dose range characteristic of Se. Additionally, the alteration of the Se valence was pivotal for conferring high biological activity and low cytotoxicity to Se‐CAP1 (Lin et al. 2023).

## 4 Conclusion

In this study, Se‐CAPs were synthesized by the microwave‐assisted approach based on the HNO_3_‐Na_2_SeO_3_ reaction. Se‐CAP1, with the highest Se content of 13.22 mg/g, was obtained with microwave power of 200 W at 70°C for 90 min. Se was connected to CAP through O‐Se‐O bonds and O‐H ··· Se bonds, increasing the molecular weight of Se‐CAP. Se‐CAP1 exhibited a significantly higher ABTS antioxidant activity compared to CAP and Na_2_SeO_3_, which might be attributed to the partial transformation of Se^4+^ to Se^0^. Notably, Se‐CAP effectively reduced the toxicity of Na_2_SeO_3_ based on the higher HT29 cell viability compared to Na_2_SeO_3_. Se‐CAP1 had a high Se release rate in gastric juice, which was assigned to a Fickian release case I transport in the SGF stage and a non‐Fickian release in the SIF stage. The study of the antioxidant activity and Se release properties of Se‐CAP in this paper is limited in vitro, and they are worth further exploration at the cellular and animal levels, focusing on the effects of Se‐CAP on the intracellular redox system and the mechanism of absorption kinetics. Overall, this study provides a reference for the optimization of the preparation of selenylation polysaccharides and a theoretical basis for the design and development of selenylation polysaccharides.

## Author Contributions

**Ruijuan Fan:** conceptualization (equal), investigation (equal), methodology (equal), writing – original draft (equal). **Wanting Dai:** conceptualization (equal), investigation (equal), methodology (equal), writing – original draft (equal). **Linqing Yue:** conceptualization (equal), investigation (equal), methodology (equal), writing – original draft (equal). **Xiaoxiao Song:** funding acquisition (equal), investigation (equal), visualization (equal), writing – review and editing (equal). **Yunpu Wang:** project administration (equal), resources (equal), supervision (equal), validation (equal). **Xian Cui:** visualization (equal), writing – review and editing (equal).

## Conflicts of Interest

The authors declare no conflicts of interest.

## Data Availability

The data will be made available on request.
